# Primary Vaginal Malignant Melanoma: A Case Report and Review of Literature

**DOI:** 10.7759/cureus.10536

**Published:** 2020-09-18

**Authors:** Mahati Paravathaneni, Vinay Edlukudige Keshava, Bohdan Baralo, Rajesh Thirumaran

**Affiliations:** 1 Internal Medicine, Mercy Catholic Medical Center, Darby, USA; 2 Hematology/Oncology, Mercy Catholic Medical Center, Darby, USA

**Keywords:** vaginal bleeding, postmenopausal, vaginal cancer, malignant melanoma

## Abstract

Primary vaginal malignant melanoma is an extremely rare and aggressive tumor with very few reported cases worldwide. It often occurs in post-menopausal women, with a mean age of 57 years. The most common presenting symptom is vaginal bleeding. Other less common presenting symptoms are vaginal discharge, vaginal mass, and pain. Vaginal melanomas are often diagnosed at an advanced stage, and despite the aggressive treatment approach, the prognosis is poor. We present to you a case of a 56-year-old post-menopausal woman who presented with intermittent vaginal bleeding and passage of dark clots. She was found to have symptomatic anemia requiring blood transfusions. Further workup revealed a mass in the upper vagina on imaging studies, and the patient eventually underwent a biopsy, which confirmed the diagnosis of malignant melanoma of the vagina on pathological examination.

## Introduction

Primary vaginal malignant melanoma is an infrequent entity, with less than 250-300 cases reported in the literature, accounting for only 0.46 cases per million women per year [1,2]. The etiology of melanoma of the female genitourinary tract, in general, has not been understood fully. However, it seems to be evident that ultraviolet radiation exposure is not a causative factor, in contrast to cutaneous malignant melanoma, given the areas are less exposed. The cell of origin has been hypothesized to arise from the melanocytes located aberrantly in the vaginal epithelium. It is a very aggressive tumor with early tumor spread and distant metastases and is usually diagnosed at an advanced stage. The overall five-year survival rate for women with vaginal melanoma is only about 8-20%, and regardless of the therapeutic modality chosen, the outcome is mostly poor. Here, we present a 56-year-old-female with primary vaginal melanoma and a review of the literature.

## Case presentation

**Figure 1:**
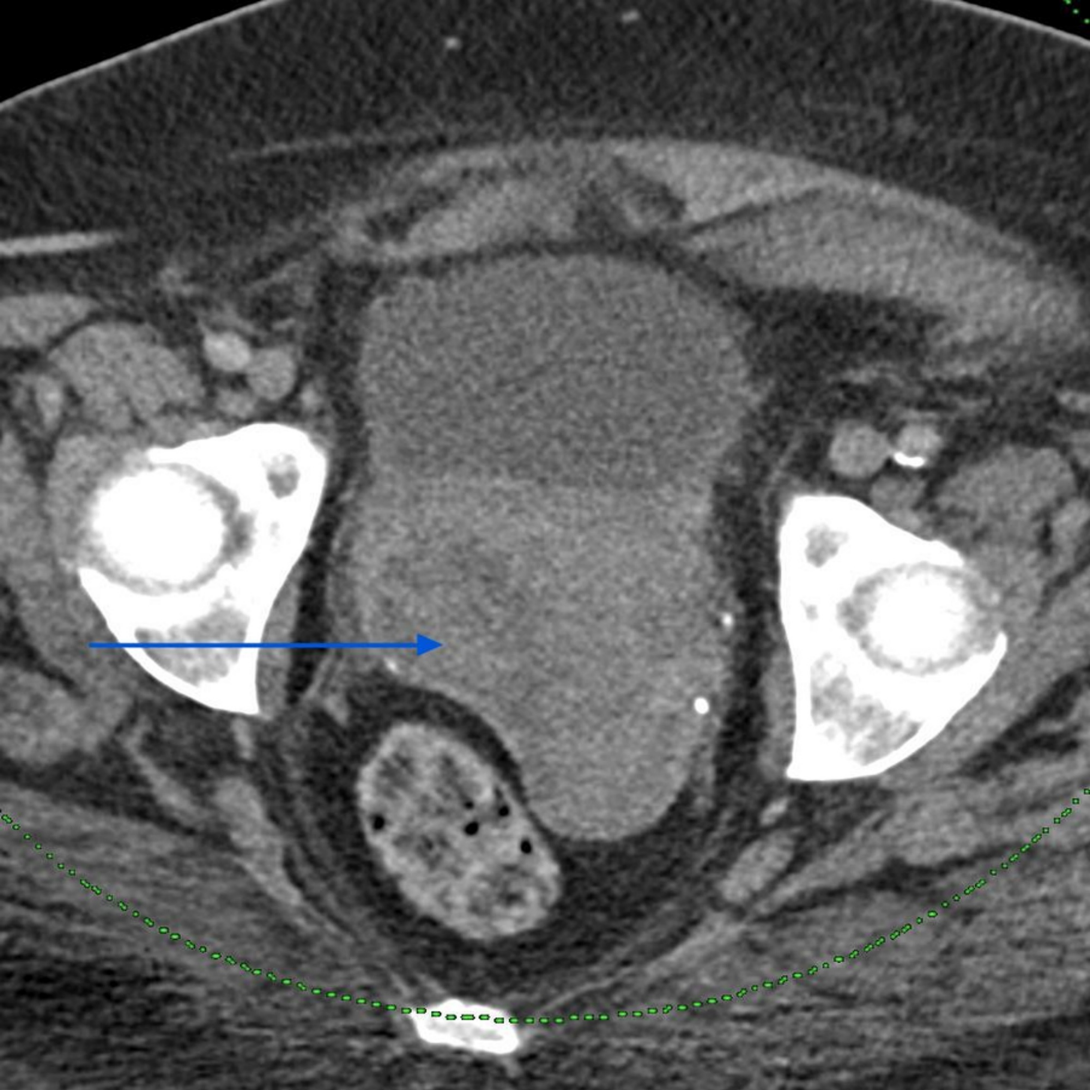
Computerized tomography scan of the abdomen and pelvis showing tumor mass involving the upper vagina - axial section (arrow)

**Figure 2:**
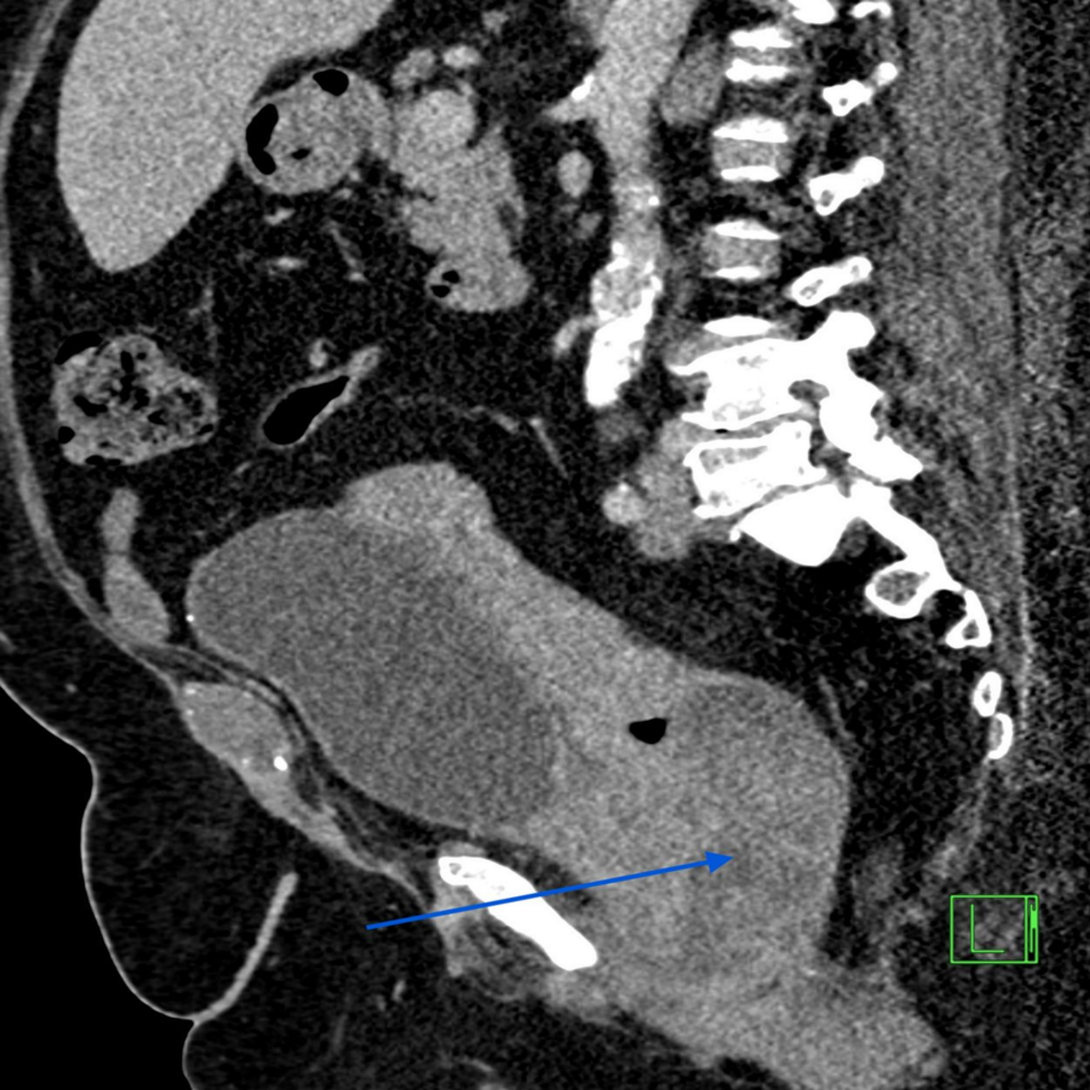
Sagittal section of the CT scan of the abdomen and pelvis showing the tumor mass in the upper vagina (arrow)

**Figure 3:**
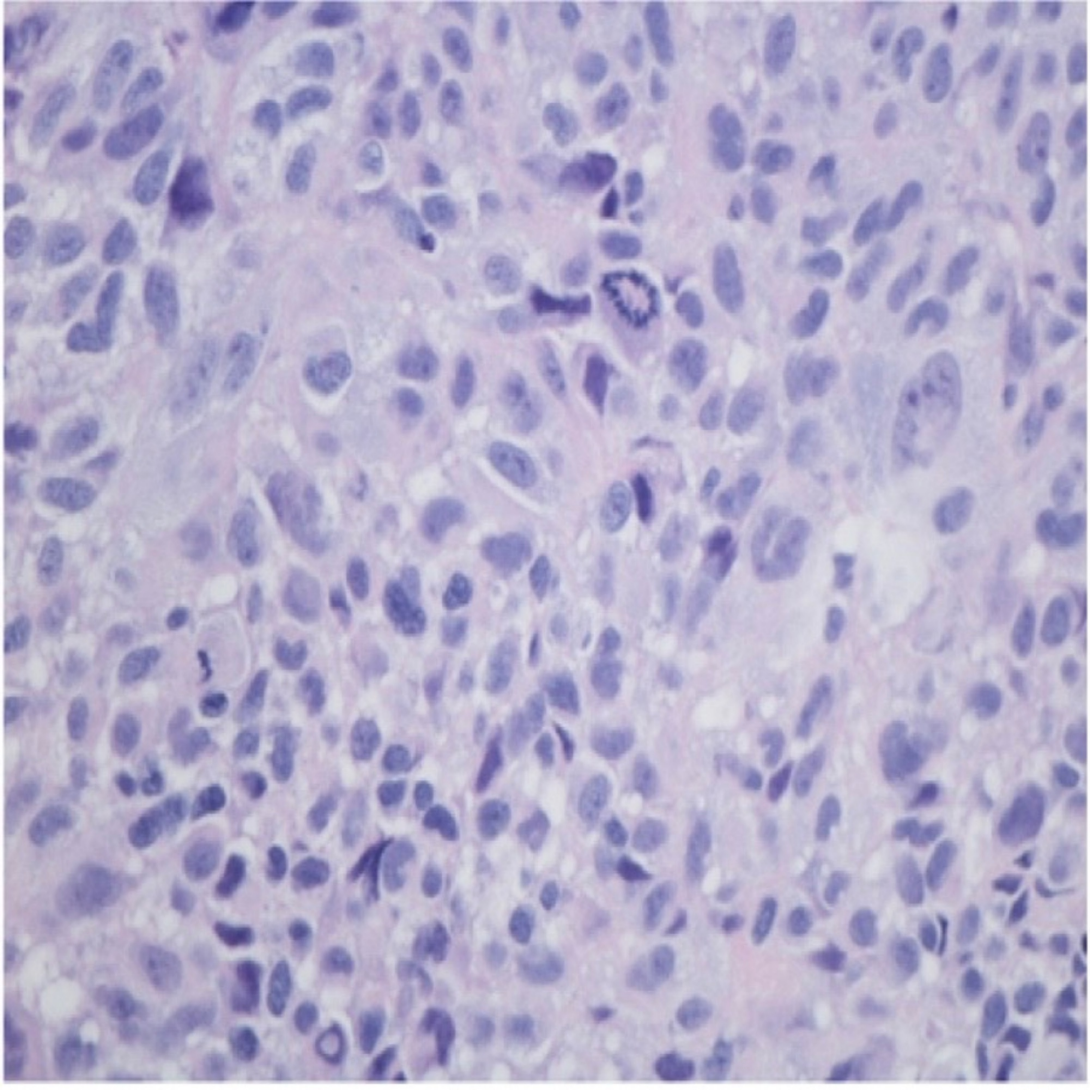
Malignant cells with nuclear pleomorphism, mitosis, eosinophilic cytoplasm, nuclear pseudo inclusion S19-4570 (H&E 400×) H&E: hematoxylin and eosin stain.

**Figure 4:**
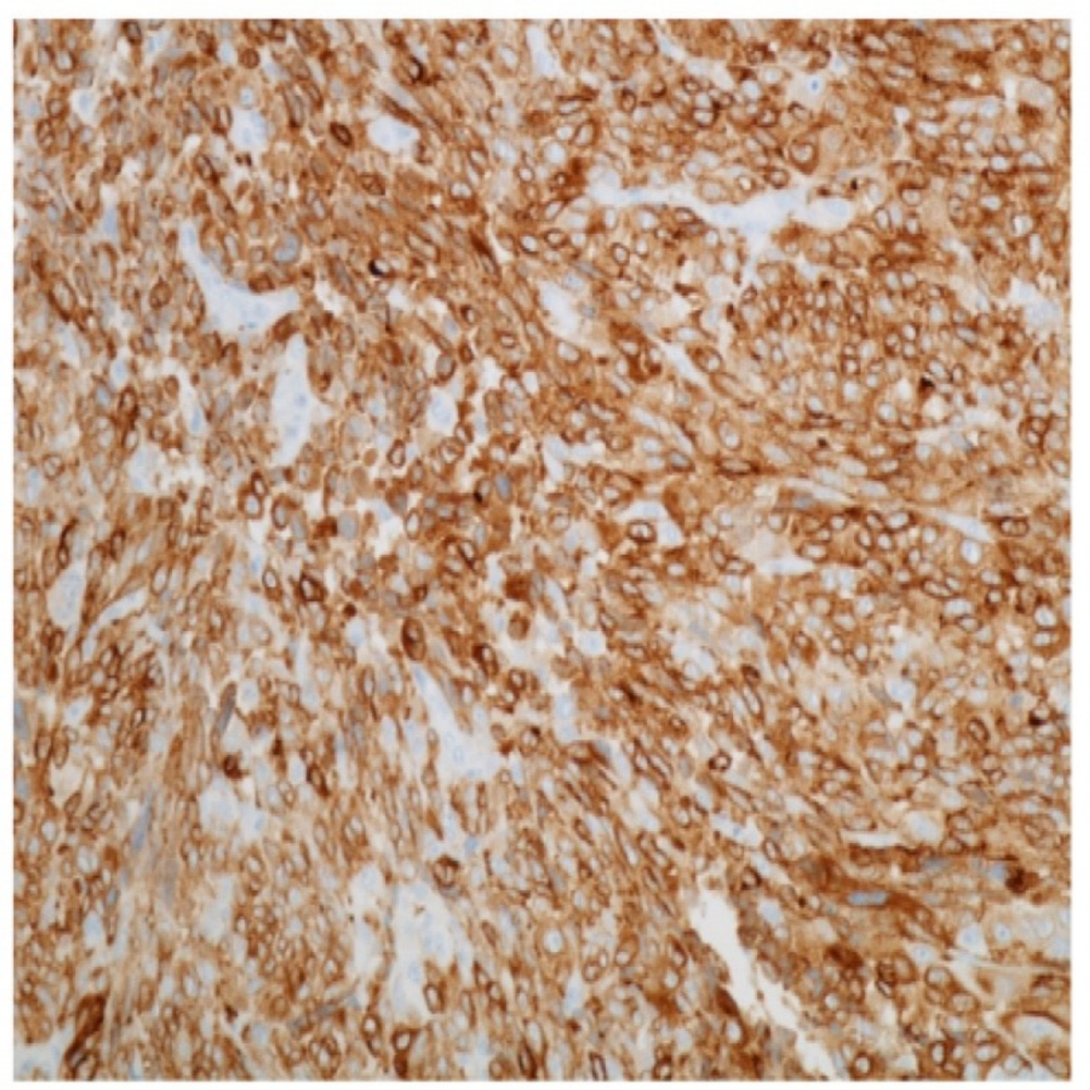
Malignant melanoma cells expressing Melan-A, 200×

A 56-year-old postmenopausal female presented with a history of several weeks of intermittent vaginal bleeding with significant passage of dark clots. The symptoms were associated with shortness of breath upon exertion, dizziness, and weakness. She had a history of three previous cesarean sections and reported being told that she had fibroids in 1994. She also had a history of heavy menstrual bleeding in her earlier cycles. On presentation, she was diagnosed with symptomatic anemia due to blood loss, with hemoglobin of 7.4 g%, and received two units of packed red blood cell transfusions. Computerized tomography scan of the abdomen and pelvis with contrast showed a large mass centered in the upper vagina compatible with vaginal or cervical malignancy along with metastatic pelvic and lower retroperitoneal adenopathy (Figures 1 and 2). She underwent a Dilatation and Curettage hysteroscopy, which showed a large friable necrotic vaginal mass measuring 8 × 4 cm^2^, consistent with malignant neoplasm of the vagina, virtually compressing the entire vaginal portion of the bladder. Biopsies were sent for the pathological examination, which confirmed the diagnosis as malignant melanoma of the vagina. Immunohistochemical stains showed tumor cells positive for S100, Melan A, vimentin, and CAM 5.2, while p63, CK5/6, cytokeratin AE1/AE3, LCA, CD34, desmin, and SMA were negative (Figures 3 and 4). The patient was medically stabilized and discharged to follow-up with an oncologist as an outpatient. However, she was eventually lost to follow-up. 

## Discussion

Primary vaginal cancers are a rare entity, accounting for less than 3% of all the diagnosed female reproductive tract cancers. Among them, the majority of them are squamous cell cancers, and malignant melanoma only accounts for about 5% of them. This makes the occurrence of primary malignant melanoma of the vagina an infrequent entity, accounting for only 0.46 cases per million women per year [1,2]. It has been established by many studies as a cancer of the elderly, postmenopausal women. This is in contrast to cutaneous melanomas, which have been shown to have an increased incidence in the younger population [2]. The most common presenting symptom is vaginal bleeding (80%), which our patient presented with too, followed by vaginal discharge (25%), palpable vaginal mass (15%), and pain (10%) [3].

There are many histological variants, epithelioid being the most common (55%), followed by less common types like spindled and mixed [3]. Due to the extensive lymphatic and vascular supply of the lamina propria of the vaginal mucous membranes, vaginal melanomas are known for their aggressive behavior with early tumor spread, local recurrences. Around 50% have lymph node metastases, and 20% have distant metastases at the time of diagnosis. The common sites of metastases are lungs, liver, bones, and brain. Tumor size is the most important prognostic factor [3-5]. The overall five-year survival rate for women with vaginal melanoma is only about 8-20% when compared to around 47% for women with melanoma of the vulva and 81% for women with cutaneous melanoma [2,6].

There are many treatment options, but none of them have been established as a single effective treatment modality. Surgical options range from as conservative as a wide local excision to as extensive as a vaginectomy plus pelvic exenteration. Still, studies have shown that the recurrence and survival rates are mostly similar [3,7]. Post-operative adjuvant radiotherapy can also be employed in cases with incomplete tumor resection or with pelvic metastases. No consistent recommendations exist when it comes to adjuvant chemotherapy. The standard of care for any stage IV melanoma is immunotherapy with Ipilimumab (anti-CTLA-4) and Nivolumab (anti-PD-1), which is the treatment our patient is likely getting [2,6]. The response rates are excellent. However, there are no studies documenting the efficacy of immunotherapy in vaginal melanoma. Agents like Dacarbazine and Interleukin-2 are used in palliative treatment, but recently, pegylated Interferon alpha-2b has shown promise in prolonging survival [5-8]. 

## Conclusions

Primary vaginal malignant melanoma is an extremely rare malignancy of the female genitourinary tract. It is a very aggressive malignancy, usually diagnosed at an advanced stage. Due to its rarity, no consistent guidelines exist with regards to the treatment. At present, the primary modality of therapy seems to be surgery with postoperative radiotherapy.
